# Vesicle-Mediated Dendritic Cell Activation in *Acinetobacter baumannii* Clinical Isolate, which Contributes to Th2 Response

**DOI:** 10.1155/2019/2835256

**Published:** 2019-12-30

**Authors:** Wei Cai, Dinesh Kumar Kesavan, Jianjun Cheng, Aparna Vasudevan, Huixuan Wang, Jie Wan, Mohamed Hamed Abdelaziz, Zhaoliang Su, Shengjun Wang, Huaxi Xu

**Affiliations:** ^1^International Genomics Research Center (IGRC), Jiangsu University, Zhenjiang 212013, China; ^2^Department of Immunology, School of Medicine, Jiangsu University, Zhenjiang 212013, China; ^3^Department of Laboratory Medicine, The Affiliated People's Hospital, Jiangsu University, Zhenjiang 212001, China

## Abstract

*Acinetobacter baumannii*, as a nonfermentation Gram-negative bacterium, mainly cause nosocomial infections in critically ill patients. With the widespread of multidrug-resistant *Acinetobacter baumannii*, the urgency of developing effective therapy options has been emphasized nowadays. Outer membrane vesicles derived from bacteria show potential vaccine effects against bacterial infection in recent study. Our present research is aimed at investigating the mechanisms involved in immune protection of mice after outer membrane vesicle immunization. As our data showed, the outer membrane vesicle from an *Acinetobacter baumannii* clinical strain could activate bone marrow-derived dendritic cells (BMDCs) to promote Th2 activity together with humoral immune responses to *Acinetobacter baumannii*-induced sepsis, which might enlighten people to have a better understanding of OMVs' role as a vaccine to prevent bacterial infections.

## 1. Introduction

*Acinetobacter baumannii* (*A. baumannii*) is Gram-negative bacteria that can cause opportunistic nosocomial infections including pneumonia, urinary tract infections, skin and soft tissue infections, and bacteremia, especially in immune-deficient patients [1]. Recently, with the application of wide spectrum of antibiotics during the clinical practice, multidrug-resistant (MDR) *A. baumannii* has spread across hospitals, predominantly in intensive care units (ICU), which has aroused public widespread attention and become a global threat to public health [2]. However, there are no effective solutions to protect patients against MDR *A. baumannii* infections yet. As a result, the existence of these MDR *A. baumannii* requires innovative clinical treatment and preventive strategies desperately, which may be considered major emergency problems worldwide.

A previous study has demonstrated that bacteria can bleb membrane vesicles from their outer membrane spontaneously and therefore these vesicles are often called outer membrane vesicles (OMVs) [3]. It is well acknowledged that OMVs are spherical bilayered vesicles ranging from 20 to 400 nm comprising abundant proteins, enzymes, virulence factors, and other biological molecules. Meanwhile, OMVs contain pathogen-associated molecular patterns (PAMPs) including lipopolysaccharide (LPS), lipids, and some outer membrane proteins [3]. OMVs can be considered having various roles in pathogenesis of bacteria, such as mediators of information exchange between bacteria and host [4] and vehicles of virulence factor transport [5]. Besides that, a recent study shows that bacterial OMVs have immunomodulatory activities, which make OMVs potential vaccine candidates for bacterial infection therapy and provide enlightened strategies for the development of antibacterial technologies [6–8]. Although there are amounts of evidences indicating that OMVs can trigger potent innate immune response in host cell and protect the host against bacterial infection [9, 10], the exact mechanisms involved in the immune response induced by OMVs remain uncertain. In the current study, we demonstrated that *A. baumannii* OMVs isolated from clinical samples promoted humoral immune response via bone marrow dendritic cell (BMDC) activation and played protective roles in *A. baumannii*-induced sepsis, which may help people broaden insights into the roles of OMVs during the antibacterial therapy.

## 2. Materials and Methods

### 2.1. Mice

Six- to eight-week-old male Balb/c mice were used in BMDC induction, and six-week-old female Balb/c mice were used in animal model establishment. These mice were purchased and maintained in the Animal Center of Jiangsu University. All animal experiments were approved by the Ethical Committee of Jiangsu University (Zhenjiang, China).

### 2.2. Bacterial Strains and OMV Purification

*A. baumannii* ATCC19606 and clinical isolate (strain JU0126) were cultured in LB broth by shaking at 37°C overnight. The ATCC19606 strain was bought from Genetimes (Shanghai, China), and the strain JU0126 was obtained from the laboratory department of the Affiliated Hospital of Jiangsu University (Zhenjiang, China). The bacterial cultures were collected and centrifuged at 8000 × *g* for 10 min at 4°C. The supernatants were filtered using a 0.45 *μ*m bottle-top vacuum filter to remove any possible bacteria. The supernatants were filtered by a 0.22 *μ*m bottle-top vacuum filter (Millipore, Cork, IRL) and then centrifuged at 4000 × *g* in millipores (100 kDa). The pellets were detained in filters and washed with sterile phosphate-buffered saline (PBS) to obtain the final solution, ultracentrifuged at 150000 × *g* for 2 h at 4°C, and resuspended in sterile PBS. The acquired OMVs were quantified using the Micro BCA Protein Assay Kit (Thermo Scientific, Massachusetts, USA) and stored at -80°C until use.

Transmission electron microscopy (TEM) was used to analyze the OMV morphology and structure. In brief, the OMVs were diluted using PBS and 10 *μ*l of the suspension was added on 300-mesh copper grids. Uranyl acetate (2%) was then dropped onto the grids to stain the OMVs. The images were captured with CM100 TEM (Philips, Netherlands).

### 2.3. BMDC Induction and OMV Stimulation

The bone marrow was taken from the femurs and tibias of the Balb/c mice. After lysing erythrocytes using ACK lysis buffer, the cells were resuspended with RPMI 1640 medium (Biological Industries) containing 10% FBS, 100 U/ml penicillin, and 100 g/ml streptomycin and seeded in a six-well plate (1 × 10^7^ cells/well) with GM-CSF (20 ng/ml) and IL-4 (10 ng/ml) (PeproTech, Rocky Hill, USA). After the cells were cultured in a 5% CO_2_ atmosphere for 2 days, the medium was changed into fresh RPMI 1640 containing GM-CSF (20 ng/ml) and IL-4 (10 ng/ml). At the fifth day, the medium was refreshed half and added with GM-CSF (10 ng/ml) and cultured for another 24 hours. The cultured cells were collected, and the purity was tested by flow cytometry. The acquired BMDCs were used in related experiments including stimulation with different doses of OMVs.

The MTT assay was applied to detect the cytotoxicity of OMVs in order to select the concentration of OMV used to stimulate BMDCs. In brief, the BMDCs were treated with different concentrations of OMVs in a 96-well plate for 20 hours. The cytotoxicity of *A. baumannii* OMVs on BMDCs was detected using the MTT Cell Proliferation and Cytotoxicity Assay Kit (Sangon Biotech, Shanghai, China). The nontoxic concentration of OMVs was determined as the stimulating concentration of BMDCs.

### 2.4. Flow Cytometry Analysis

4 × 10^5^ BMDCs were cocultured with 10 *μ*g/ml OMVs at 37°C in an atmosphere containing 5% CO_2_ for 20 hours in a 24-well plate. Then, the cells were washed with PBS and stained with anti-CD11c-fluorescein isothiocyanate, anti-CD86-phycoerythrin, anti-CD80-fluorescein isothiocyanate, and anti-MHC-II-phycoerythrin (cat nos. 117305, 105106, 104705, and 107608, BioLegend, CA, USA). Labeled cells were washed with PBS and detected using a FACSCalibur flow cytometer (BD Biosciences, San Jose, CA) with CellQuest software version 3.2 (BD Biosciences, San Jose, CA). The results were analyzed by FlowJo software version 10.

To detect intracellular cytokine, the splenocytes were suspended with RPMI 1640 containing 10% FBS and cocultured with 2 *μ*g/ml anti-CD3 and anti-CD28 antibodies (cat nos. 100208 and 102112, BioLegend, CA, USA) at 37°C for 12 hours in a 5% CO_2_ humidified incubator. After the stimulation with two kinds of antibodies, the cells were stained with anti-CD4-fluorescein isothiocyanate (cat no. 130308, BioLegend, CA, USA) and then incubated with anti-IFN-*γ*-allophycocyanin, anti-IL-17A-phycoerythrin, and anti-IL-4-PerCP-Cyanine5.5 (cat nos. 505810, 506904, and 504124, BioLegend, CA, USA), respectively. The labeled cells were washed with PBS, and flow cytometry analysis was performed.

### 2.5. Quantitative RT-PCR

BMDCs were collected after OMV simulation, and total RNA was extracted by Invitrogen TRIzol (Thermo Scientific, Massachusetts, USA). 1000 ng RNA was reverse transcribed using a PrimeScript® RT reagent kit (Takara Bio, Japan) according to the manufacturer's protocols at 37°C for 15 min and 85°C for 5 sec. Primers used in the present study were designed and synthesized by Sangon Biotech. The sequences of primers are listed in Table 1. Quantitative PCR was conducted in the StepOnePlus Real-Time PCR System (Applied Biosystems) and SYBR® Premix Ex Taq™ (Takara) based on the manufacturer's protocols. Fold changes were calculated according to the cycle threshold value compared with the expression of *β*-actin. All samples were tested in triplicate.

### 2.6. ELISA

The serum IgM and IgG and the cytokines TNF-*α* and IL-4 were measured with the ELISA kit according to the manufacturer's protocols (Multisciences, Hangzhou, China). All samples were tested in triplicate, and the concentration was acquired from a standard curve.

### 2.7. Evaluation of Immunopromotive and Protective Effects Induced by *A. baumannii* OMVs in Mice

To determine the lethal dose of *A. baumannii* infection in mice, different doses of 200 *μ*l *A. baumannii* of the two stains were intravenously injected into six-week-old female Balb/c mice and the survival rates were monitored every 12 hours for three days. According to the results of survival rate curves, the lethal dose (OD_600_ = 1.5 for ATCC19606 and OD_600_ = 1.75 for the strain JU0126) of *A. baumannii* was confirmed.

To prepare the immunization of OMVs, 600 *μ*l OMVs (50 *μ*g/ml) was premixed with 600 *μ*l Imject® Alum adjuvant (40 mg/ml) (Thermo Scientific, Massachusetts, USA) and mixed well for 30 minutes according to the manufacturer's protocols. In order to evaluate the effects of OMV immune protection in vivo, mice were intramuscularly immunized with 200 *μ*l *A. baumannii* OMVs mixed with adjuvant for three weeks at 1-week intervals. As a control, mice were injected with sterile PBS instead of OMVs. The immunized mice were sacrificed three days after the last immunization, and the spleen was obtained for analyzing immune function of splenic lymphocytes. Meanwhile, the mice were injected intramuscularly with *A. baumannii* OMVs for the same time of period and challenged with a lethal dose of the two strains of *A. baumannii* seven days after the last immunization. The survival rate was monitored every 12 hours for 3 days.

### 2.8. Statistical Analysis

All data were depicted as means ± standard deviation (SD). The statistically significant differences between groups were determined by analysis of variance (ANOVA) or Student's *t*-test using Prism software (GraphPad, San Diego, USA). *p* < 0.05 was considered significant.

## 3. Results

### 3.1. Characterization of OMVs Released by *A. baumannii*

**Figure 1:**
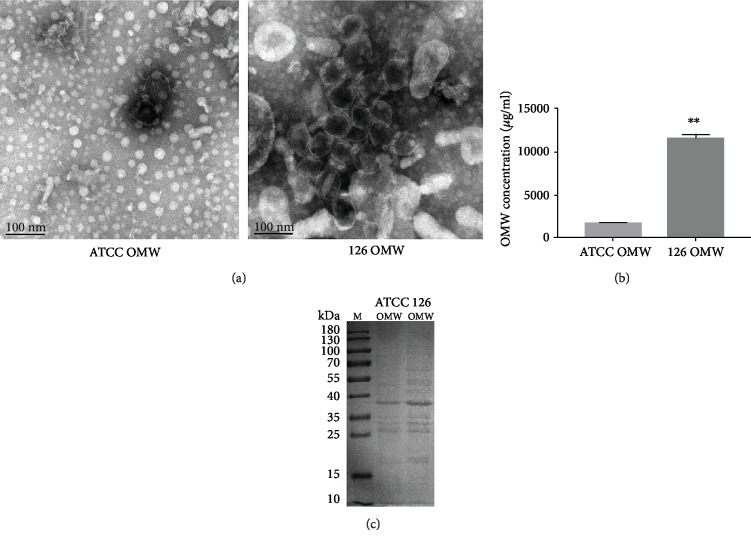
Characterization of OMVs released by *A. baumannii.* (a) Transmission electron micrograph images of *A. baumannii* ATCC19606-derived OMVs (left) and clinically isolated strain JU0126-derived OMVs (right). Scale bars indicate 100 nm. (b) Quantitative detection of proteins in OMVs from *A. baumannii* ATCC19606 and clinical strain JU0126 by the BCA assay. ^∗∗^*p* < 0.01. (c) Distribution of proteins in OMVs (*A. baumannii* ATCC19606 and JU0126) by SDS-PAGE.

TEM analysis revealed the morphology and structure of OMVs produced by the *A. baumannii* standard strain (ATCC19606) and clinical isolate (strain JU0126) (Figure 1(a)). Both the strain ATCC19606 and the strain JU0126 could secrete spherical nanosized lipid-bilayered vesicles. Meanwhile, the results of protein spectrum showed that the OMVs produced by the strain JU0126 were higher than those produced by the strain ATCC19606 (Figures 1(b) and 1(c)), which may provide the evidences that the clinical strains can produce more OMVs due to increased vitality of bacteria in human internal environment.

### 3.2. *A. baumannii*-Derived OMVs Upregulate the Expression of Costimulatory Molecules and MHC-II in BMDCs

**Figure 2:**
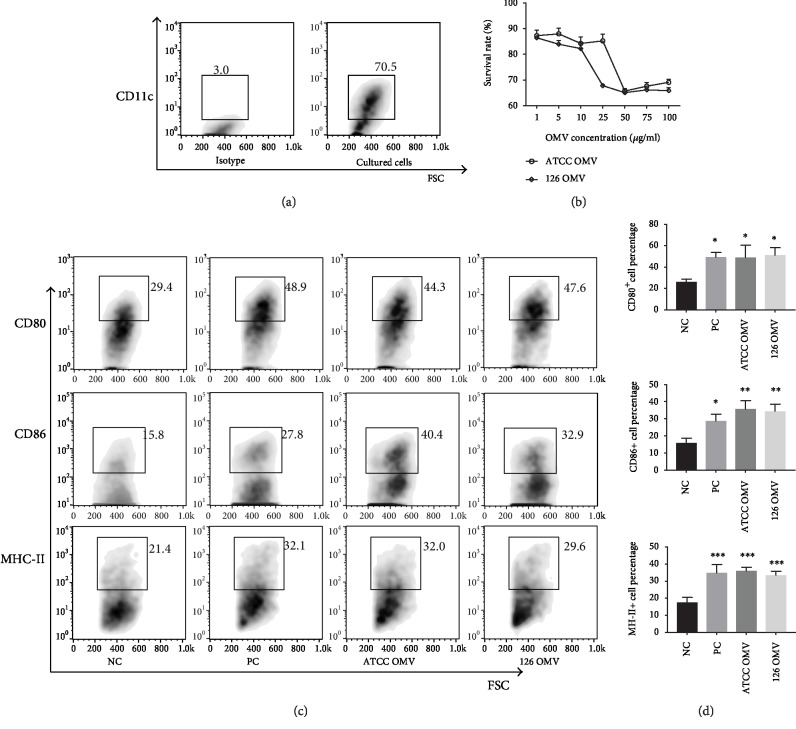
*A. baumannii* OMVs can activate BMDCs by upregulation of costimulatory molecules and MHC-II. (a) Purity determination of bone marrow-derived dendritic cells (BMDCs) measured by flow cytometry. (b) Analysis of cytotoxicity of OMVs on BMDCs. The different dilutions of OMVs were cocultured with BMDCs for 20 h to observe the cell survival. 10 *μ*g/ml OMVs was selected as the optimum concentration for stimulating BMDCs. Data were presented as mean ± SD (*n* = 3 per group). (c) Flow cytometry analysis of the expression of costimulatory molecules and MHC-II on the BMDCs after OMV treatment. (d) Statistical analysis for the results of flow cytometry showed that the expression levels of CD80, CD86, and MHC-II increased significantly. (*n* = 3 per group, ^∗^*p* < 0.05, ^∗∗^*p* < 0.01, and ^∗∗∗^*p* < 0.001). NC: negative control; PC: positive control.

Flow cytometry analysis was used to estimate the frequency of CD11c^+^ cells isolated from mouse bone marrow. It reveals that the purity of BMDCs is about 70% (Figure 2(a)). 5 × 10^4^ BMDCs were cocultured with different concentrations of OMVs ranging from 1 *μ*g/ml to 100 *μ*g/ml in a 96-well plate. As shown in Figure 2(b), the high concentration of OMVs could induce cytotoxicity to BMDCs, while less than 10 *μ*g/ml OMVs did not affect cell survival. 10 *μ*g/ml OMVs was selected as the appropriate concentration for stimulating BMDCs. The results also provided indirect evidences that the BMDCs were more susceptible to OMVs derived from the clinical strain compared with OMVs produced by the strain ATCC19606. Based on the previous study, we used these two different OMVs to be cocultured with BMDCs for 20 h (LPS as a positive control) and evaluated the activation of BMDCs. Our data showed that *A. baumannii* OMVs could promote BMDCs to express more CD80, CD86, and MHC-II (Figures 2(c) and 2(d)), which indicated that *A. baumannii* OMVs might contribute to enhancing antigen presenting ability and triggering innate immune response in BMDCs. *A. baumannii* OMVs may have potential to stimulate adaptive immune response in the host via activating BMDCs.

### 3.3. Enhanced Expression of Th2 Cytokines and Inflammatory Factors in BMDCs Induced by *A. baumannii* OMVs

**Figure 3:**
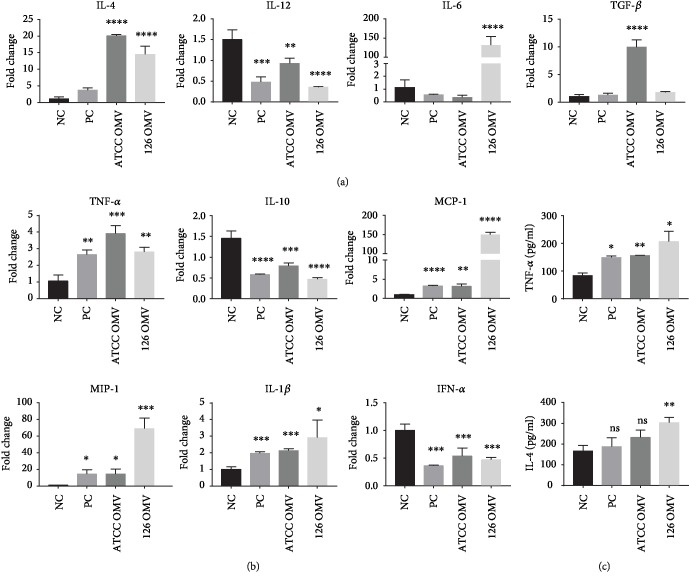
Expression levels of cytokines involving in T cell differentiation by OMV-stimulated BMDCs. The expression of cytokines in 10 *μ*g/ml OMV-induced BMDCs. (a, b) The results from IL-1*β*, IL-4, IL-6, IL-10, IL-12, TGF-*β*, MIP-1, and TNF-*α* expression detected by qRT-PCR. (c) The level of TNF-*α* and IL-4 was assessed by ELISA. Data are shown as mean ± SD (*n* = 3 per group, ^∗^*p* < 0.05, ^∗∗^*p* < 0.01, ^∗∗∗^*p* < 0.001, and ^∗∗∗∗^*p* < 0.0001). NC: negative control; PC: positive control.

Dendritic cells are important antigen-presenting cells. They are the first barrier to immune stimulation by foreign antigens and play a dual role in innate immunity and adaptive immune response. To investigate the immunostimulatory effect of *A. baumannii*-derived OMVs, BMDCs were coincubated with 10 *μ*g/ml OMVs for 20 hours and the expression levels of related cytokines were analyzed via qRT-PCR. Our data showed that the OMVs derived from *A. baumannii* could promote BMDCs to produce more IL-4 (Figure 3(a)), which might contribute to Th2 polarization. *A. baumannii* OMVs also elicit potent innate immune responses presented as an increment in the production of inflammatory factors such as IL-1*β* and TNF-*α* and excretion of chemokines like monocyte inflammatory protein-1*α* (MIP-1*α*) and monocyte chemotactic protein-1*α* (MCP-1*α*). Meanwhile, IL-10, a well-known anti-inflammatory cytokine, together with interferon *α* (IFN-*α*) was decreased (Figure 3(b)). Similar results were found in ELISA for the expression of inflammatory cytokines (Figure 3(c)). However, we found that these two different OMVs show discrepancies in stimulating BMDCs to secret IL-4. IL-4 production can be observed through the obvious increment in the strain JU0126-derived OMV group but slight increment in the strain ATCC19606-derived OMV group (Figure 3(c)). These findings suggest that *A. baumannii*-derived OMVs may have vaccine potential to induce innate and adaptive immunity.

### 3.4. *A. baumannii*-Derived OMVs Stimulate Th2 Response and Promote Antibody Production *In Vivo*

**Figure 4:**
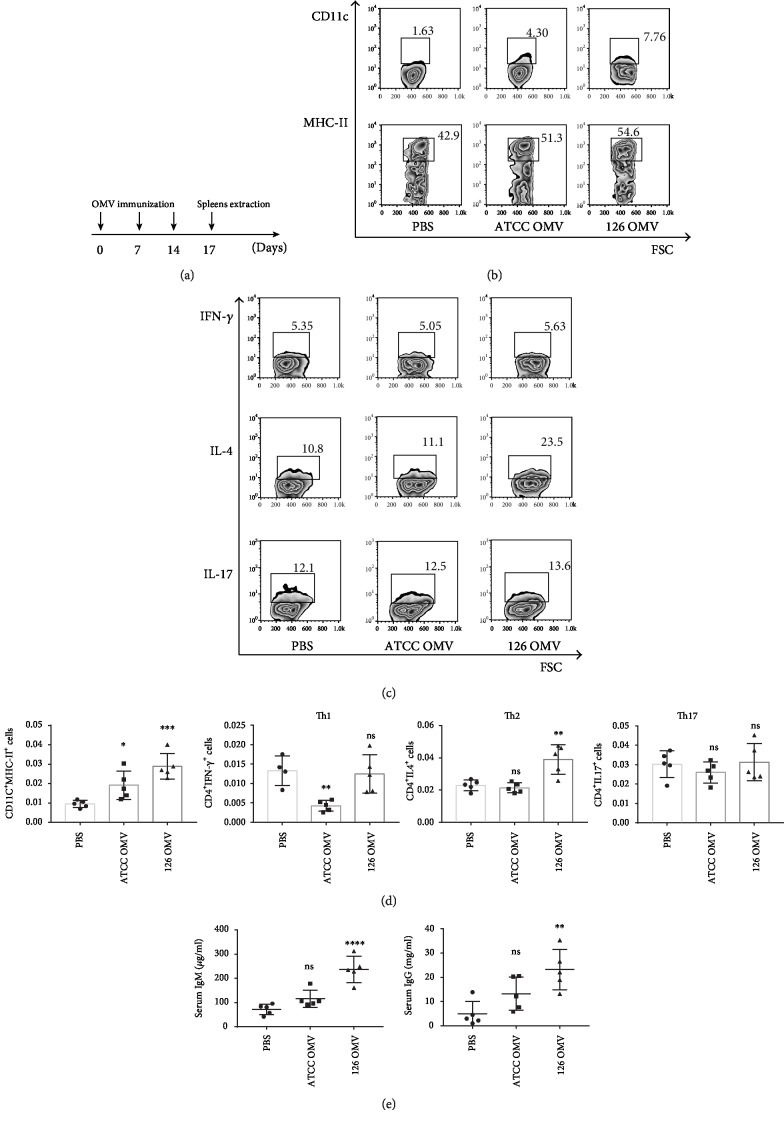
Effects of *A. baumannii* OMV immunization in vivo. (a) The process of OMV immunization and spleen extraction for the following related analysis. OMVs and adjuvant control were injected intramuscularly once a week for three weeks, and then, the mice were sacrificed for related analysis three days after the last immunization. (b) Flow cytometry analysis for frequency of activated dendritic cells in the spleen after OMV immunization in vivo. (c) Intracellular cytokine assay for the percentages of IFN-*γ*, IL-4, and IL-17 in splenic CD4^+^ T cells, which were stimulated with anti-CD3 and anti-CD28 antibodies. (d) Statistical analysis of CD11c^+^MHCII^+^ cells, CD4^+^IFN-*γ*^+^ cells, CD4^+^IL-4^+^ cells, and CD4^+^IL-17^+^ cells in splenocytes after OMV immunization in vivo. (e) Levels of serum IgM and IgG in OMV-immunized mice and sham-immunized mice. Data obtained from at least three independent experiments (^∗^*p* < 0.05, ^∗∗^*p* < 0.01, ^∗∗∗^*p* < 0.001, and ^∗∗∗∗^*p* < 0.0001). ns indicates no significance.

*A. baumannii*-derived OMVs (10 *μ*g/mice) were intramuscularly injected into mice according to the scheme in Figure 4(a), the splenocytes were obtained to analyze the percentages of dendritic cells and some CD4+ T cells including Th1, Th2, and Th17, and the serum was isolated to detect the levels of immunoglobulins (IgG and IgM) and bacterial agglutination. The results showed that the percentages of MHC-II^+^CD11c^+^ dendritic cells and IL-4^+^CD4^+^ Th2 cells were significantly enhanced in the mice injected with strain JU0126-derived OMVs (Figures 4(b) and 4(c)), but there was no significant change in the percentages of IL-17^+^CD4^+^ cells and IFN-*γ*^+^CD4^+^ cells from mice injected with strain JU0126- or ATCC19606-derived OMVs (Figures 4(c) and 4(d)). In addition, the OMV immune serum mixed with two strains of *A. baumannii* showed obvious agglutination, the agglutination rate was 1 : 32, and the levels of serum IgG and IgM were obviously increased in mice injected with strain 126-derived OMVs but not observed in strain ATCC19606-derived OMVs (Figure 4(e)). Our data suggest that the clinical isolation strain JU0126-derived OMVs can elicit efficient Th2 responses via activating DCs.

### 3.5. Protective Effect of *A. baumannii*-Derived OMV Pretreatment on Mice Infected with *A. baumannii*

**Figure 5:**
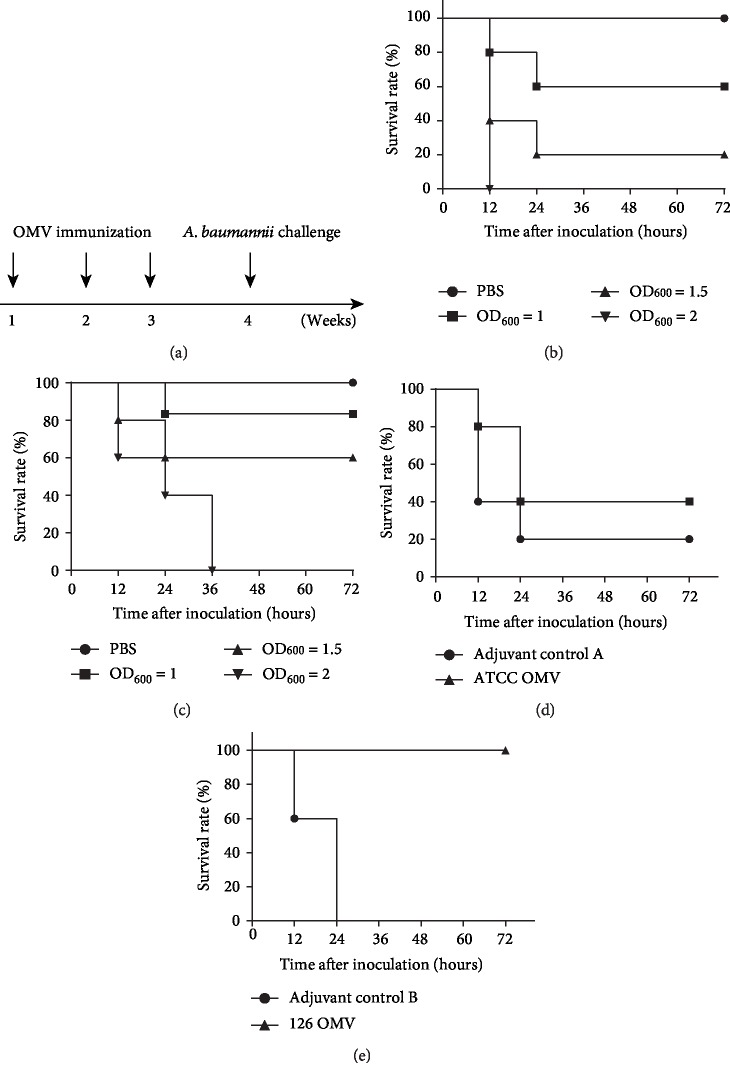
Evaluation of the survival rate against *A. baumannii*-induced sepsis after OMV immunization in mice. (a) The programme of OMV immunization and bacterial challenges. OMVs were immunized as previously described, and then, a lethal dose of *A. baumannii* was injected intravenously into immunized mice together with sham-immunized mice. The survival rate was monitored every 12 hours for three days after *A. baumannii* challenge. (b, c) Determination of the lethal dose of *A. baumannii* ATCC19606 or strain JU0126 in mice. (d, e) Survival rates of immunized mice challenged with *A. baumannii* ATCC19606 (OD_600_ = 1.5) and strain JU0126 (OD_600_ = 1.75). All data are obtained from at least three independent experiments.

As depicted in Figure 5(a), after pretreatment of *A. baumannii*-derived OMVs, a mouse model of *A. baumannii* infection was established by intravenous injection with different amounts of bacteria, and the results are shown in Figures 5(b) and 5(c). Briefly, the amounts of the strain JU0126 (OD_600_ = 1.75) and the strain ATCC19606 (OD_600_ = 1.5) were used in this experiment. The mice were immunized intramuscularly with two different *A. baumannii* OMVs, respectively, every week for 3 weeks; then, the mice were challenged with a lethal dose of *A. baumannii* after the last OMV immunization, and the survival rate was monitored every 12 hours for 3 days. Our data shows that strain JU0126-derived OMVs could improve the survival rate of mice with *A. baumannii*-induced sepsis (Figure 5(e)), but the strain ATCC19606-derived OMVs had no obvious effect (Figure 5(d)). It indicates that *A. baumannii*-derived OMVs may act as a vaccine candidate to prevent sepsis caused by *A. baumannii* infection.

## 4. Discussion

**Figure 6:**
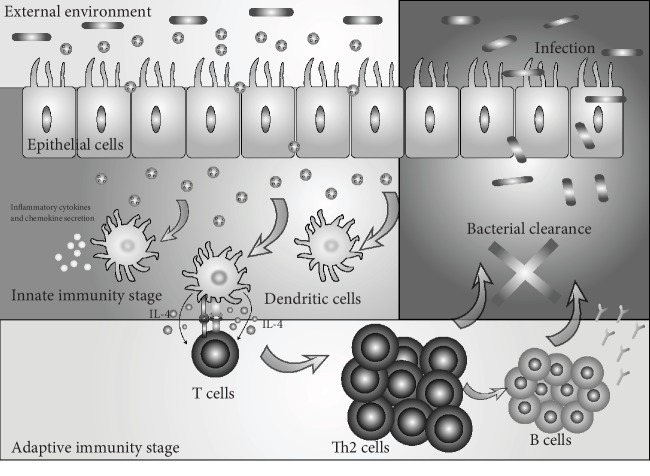
Mechanisms of *A. baumannii*-derived OMVs act as defenders against bacterial infection. *A. baumannii*-derived OMVs can promote dendritic cells to produce related inflammatory cytokines or chemokines at the innate immunity stage. When it comes to the adaptive immunity stage, OMV-activated dendritic cells mediate T cell polarization via binding with naive T cells, and the cytokines produced by dendritic cells contribute to Th2 polarization, which leads to specific humoral immune responses to bacterial infection in the host.

It is not the first time for bacterial OMVs to be considered potential vaccine candidates against bacterial infection. Previous studies have shown that OMVs derived from bacteria have protective efficacy to improve the survival rate of infection models when they are immunized in advance [11, 12]. In the present study, we aim to find evidences about the defensive mechanisms involved in dendritic cells, well-known professional antigen-presenting cells (APCs), and their relationships with adaptive immune cells such as T and B cells in the model of OMV immunization. Besides that, we examined the effects of *A. baumannii* OMVs via coincubation with BMDCs in vitro and analyzed the protective roles of *A. baumannii*-derived OMVs in *A. baumannii*-induced sepsis in vivo. These findings implicate that the clinical isolation *A. baumannii*-derived OMVs are efficient in the prevention of *A. baumannii* infection via activating dendritic cells to induce potent innate and adaptive immune responses (Figure 6).

*A. baumannii* is increasingly becoming an opportunistic pathogen threatening public health. As a common bacterium causing nosocomial infections, especially pneumonia and bloodstream infection, *A. baumannii* has been listed as one of the major clinical challenges because of its high morbidity and mortality in hospitals [13]. Recently, many reports show that *A. baumannii* have a high rate of antibiotic resistance, which may aggravate the already harsh situations against *A. baumannii* infection [14, 15]. As a result, a practical and effective treatment is imperative for the prevention of *A. baumannii* infection.

OMVs were discovered many years ago. Initially, they were considered bypass products during the growth of the parent bacterium. But until now, more and more researches have revealed that OMVs may be important in the communication between bacteria, treated as vehicles for bacterial pathogenicity, and correlative to immunity against bacterial infections [16–18]. Indeed, our previous study has demonstrated that OMVs contain numerous proteins, virulence factors, and something else, which make OMVs a potential vaccine candidate to elicit specific immune responses in the host. In the present study, we report that OMVs from a clinical isolation strain of *A. baumannii* named JU0126 have protective effects on the sepsis model and elucidates mechanisms involved in these types of protection. What is unavoidable is that *A. baumannii* ATCC19606-derived OMVs fail to show anticipated effects, which may be attributed to different complicated proteins inside OMVs that may possibly influence the host immune responses, and further analysis of complex OMV components is needed. Based on the previous results performed by our group using mass spectrum (data not shown), OMVs derived from the clinical isolation of *A. baumannii* contain different types or amounts of protein compared with those derived from the strain ATCC19606, which may be responsible for the discrepancies of Th2 induction. For example, the chaperone homologous protein HscA, putative acriflavine resistance protein A, and the signal recognition particle receptor FtsY can be found in OMVs derived from the ATCC strain, but these types of protein can hardly be detected in OMVs from the JU0126 strain. Besides that, the YqaJ viral recombinase family protein, outer membrane porin (OprD family), and type IV pilus biogenesis/stability protein PilW were expressed in high amounts in JU0126 strain-derived OMVs compared with those from the ATCC strain. Overall, because of numerous complicated components inside OMVs, further exploration is required to explain the discrepancies about immune responses.

It is well acknowledged that antibody secretions and adaptive immunity play critical roles in host defenses toward bacterial infections including *A. baumannii* [19]. The same as OMV immunization, adoptive transfer of serum or splenocytes after OMV immunization has similar protective effects against bacterial infection in mice [20, 21]. However, the exact immune responses to OMV immunization seem indefinite. A more acceptable statement is that the immune responses elicited by bacterial OMVs involve the mixtures of cellular and humoral immunity [22, 23]. In addition, OMV immunization of different origins even from allogeneic species has discrepancy in types of host immunity, which may be attributed to different components carried by OMVs [24]. Different from other bacteria-derived OMVs, our present findings indicated that immunization with OMVs derived from a clinical isolation of *A. baumannii* prevented the spread of bacteria mainly via both antibodies and IL-4-mediated immune responses.

To the best of our knowledge, although the protective bonus [19] and potent innate immune response [10] have been reported elsewhere, this is the first study reporting that *A. baumannii* OMVs can activate dendritic cells to promote Th2 and humoral immune responses in the host, which may provide evidences and inspirations for the possibility of utilizing OMVs as vaccine to prevent *A. baumannii* infection. Next research should focus on which components carried by OMVs are responsible for the protection against *A. baumannii*; despite the fact that numerous work remains to be explored, we have great confidence in clinical implications of OMVs or derivatives in the near future.

## Figures and Tables

**Table 1:** Details of the related primers used in this study.

Gene	Forward (5′^_^3′)	Reverse (5′^_^3′)	Product size
IFN-*α*	TGGCGGTGCTGAGCTACTGG	TGTACCAGGAGTGTCAAGGCTCTC	97 bp
TNF-*α*	ATGTCTCAGCCTCTTCTCATTC	GCTTGTCACTCGAATTTTGAGA	179 bp
IL-12	GACCTGTTTACCACTGGAACTA	GATCTGCTGATGGTTGTGATTC	211 bp
IL-6	CTCCCAACAGACCTGTCTATAC	CCATTGCACAACTCTTTTCTCA	97 bp
IL-4	TACCAGGAGCCATATCCACGGATG	TGTGGTGTTCTTCGTTGCTGTGAG	139 bp
IL-10	TTCTTTCAAACAAAGGACCAGC	GCAACCCAAGTAACCCTTAAAG	81 bp
*β*-Actin	CTACCTCATGAAGATCCTGACC	CACAGCTTCTCTTTGATGTCAC	90 bp
Tgfb	CCAGATCCTGTCCAAACTAAGG	CTCTTTAGCATAGTAGTCCGCT	169 bp
IL-1*β*	TCGCAGCAGCACATCAACAAGAG	TGCTCATGTCCTCATCCTGGAAGG	118 bp
MIP-1*α*	TTGCTGTTCTTCTCTGTACCAT	AATAGTCAACGATGAATTGGCG	129 bp
MCP-1	TTTTTGTCACCAAGCTCAAGAG	TTCTGATCTCATTTGGTTCCGA	101 bp

## Data Availability

The data used to support the findings of this study are available from the corresponding author upon request.
